# An Integrated Approach to Unravel Hidradenitis Suppurativa Etiopathogenesis

**DOI:** 10.3389/fimmu.2019.00892

**Published:** 2019-04-25

**Authors:** Paola M. Tricarico, Michele Boniotto, Giovanni Genovese, Christos C. Zouboulis, Angelo V. Marzano, Sergio Crovella

**Affiliations:** ^1^Department of Advanced Diagnostics, Institute for Maternal and Child Health-IRCCS “Burlo Garofolo”, Trieste, Italy; ^2^University of Paris Est-Créteil and INSERM U955/IMRB-Team 16, Créteil, France; ^3^UOC Dermatologia, Fondazione IRCCS Ca' Granda Ospedale Maggiore Policlinico, Milan, Italy; ^4^Dipartimento di Fisiopatologia Medico-Chirurgica e Dei Trapianti, Università degli Studi di Milano, Milan, Italy; ^5^Departments of Dermatology, Venereology, Allergology and Immunology, Dessau Medical Center, Brandenburg Medical School Theodor Fontane, Dessau, Germany

**Keywords:** hidradenitis suppurativa, genomics, transcriptomics, proteomics, OMICs, data integration, public repositories

## Abstract

Hidradenitis suppurativa/acne inversa (HS) is a chronic inflammatory disease involving hair follicles that presents with painful nodules, abscesses, fistulae, and hypertrophic scars, typically occurring in apocrine gland bearing skin. Establishing a diagnosis of HS may take up to 7 years after disease onset. HS severely impairs the quality of life of patients and its high frequency causes significant costs for health care system. HS patients have an increased risk of developing associated diseases, such as inflammatory bowel diseases and spondyloarthropathies, thereby suggesting a common pathophysiological mechanism. Familial cases, which are around 35% of HS patients, have allowed the identification of susceptibility genes. HS is perceived as a complex disease where environmental factors trigger chronic inflammation in the skin of genetically predisposed individuals. Despite the efforts made to understand HS etiopathogenesis, the exact mechanisms at the basis of the disease need to be still unraveled. In this review, we considered all OMICs studies performed on HS and observed that OMICs contribution in the context of HS appeared as not clear enough and/or rich of useful clinical information. Indeed, most studies focused only on one aspect—genome, transcriptome, or proteome—of the disease, enrolling small numbers of patients. This is quite limiting for the genetic studies, from different geographical areas and looking at a few aspects of HS pathogenesis without any integration of the findings obtained or a comparison among different studies. A strong need for an integrated approach using OMICs tools is required to discover novel actors involved in HS etiopathogenesis. Moreover, we suggest the constitution of consortia to enroll a higher number of patients to be analyzed following common and consensus OMICs strategies. Comparison and integration with the findings present in the OMICs repositories are mandatory. In a theoretic pipeline, the Skin-OMICs profile obtained from each HS patient should be compared and integrated with repositories and literature data by using appropriate InterOMICs approach. The final goal is not only to improve the knowledge of HS etiopathogenesis but also to provide novel tools to the clinicians with the eventual aim of offering a tailored treatment for HS patients.

## Introduction

Hidradenitis suppurativa/acne inversa (HS) is a chronic-recurrent, inflammatory, debilitating skin disease that usually presents after puberty. It is hallmarked by painful, deep-seated, chronic, suppurating lesions most commonly located in the axillary, inguinal, anogenital, and infra-mammary areas (1, 2). Treatment strategies rely on both medical and surgical options. Medical treatment is founded on the use of antibiotics, such as tetracyclines, rifampicin and clindamycin, retinoids, and immunosuppressive agents. Anti-TNFα agents, notably adalimumab that is the only biologic agent approved for HS, are the mainstay of treatment in moderate-to-severe HS (3–5).

HS incidence in different countries ranges from 6 per 100.000 in Olmsted County (6) to 6.7 per 1,000 in Australia (7) to 1.8 per 100 in Denmark (8). This epidemiological variability may reflect differences both in the awareness of physicians and in susceptibility to HS in distinct populations. In fact, it has been shown that in the United States, African Americans are more susceptible to HS, even if the underlying causes are unknown (9, 10).

The idea that the disorder is primarily caused by an inflammation of apocrine sweat glands is nowadays rejected and follicular hyperkeratosis and perifolliculitis are regarded as the earliest events detected in HS skins (11, 12). Follicular hyperkeratosis probably engenders the occlusion of the terminal hair follicles, its dilation, and finally its rupture (12). It is thought that keratin, corneocytes, hair shaft, sebum products spilled from breached pilosebaceous units into the dermis (13) can act as danger-associated molecular patterns (DAMPs) activating an immune response in deep dermis sustained by CD3+ T cells (mainly CD4+, but also CD8+), B lymphocytes, macrophages and, more importantly, neutrophils (13). CD4+ T cells (T helper (Th)) and neutrophils are the main producers of IL-17 (14, 15) that, together with TNF-α, IL-1β, and IL-10, are the cytokines found consistently overexpressed in HS lesional and perilesional skin (16–19).

Very few data are available for the events of the “subclinical inflammation” phase (20) but the hypothesis of microfilm-forming microbes or skin pathogens as main drivers of HS inflammation is fading away. In fact, Ring et al. (21) showed by peptide nucleic acid (PNA)-FISH a paucity rather than an enrichment of bacterial aggregates in HF pre-clinical HS skin when compared with healthy controls. Next-generation sequencing (22) studies performed on skin microbiome of HS patients during flares showed the existence of a dysbiosis (21, 23) that could allow the development of a pathobiome or an augmented expression of virulence factors by otherwise harmless commensal bacteria (24, 25) probably driven by host inflammation, as shown in atopic dermatitis (26). It is still debated whether these bacteria maintain a vicious circle that amplifies and sustains skin inflammation or are the *primum movens* of the disease (27).

## Genomics

### Genetics of HS: γ-Secretase

Identification of English families where HS was transmitted as an autosomal dominant trait has shed light on the genetic basis of disease susceptibility (28). Still, in pedigrees with members from more generations affected, the percentage of first-degree relatives affected was 34%. This was, according to the authors, quite far from the 50% expected for a dominant disease but was incompatible with a multigenic trait transmission. Interestingly, some families showed more women affected than men, with a 3:1 female to male ratio that today is confirmed by several epidemiological studies (8, 9), whilst other ones showed a preferential male-to-male transmission predicting that one gene-one disease cannot be applied for HS. Authors stated that assessment of genetic transmission could have been complicated by reduced penetrance, unpredictable onset age, and variable clinical severity, leading to the fact that family members presenting mild clinical manifestations might have remained undiagnosed. In addition, a strong feeling of shame associated with the disorder may lead relatives to conceal their condition to the family (28).

Gao and colleagues analyzed a four generations Chinese family by linkage analysis using microsatellite markers mapping the genes for HS in a region of about 76 Mb at chromosome 1 (1p21.1 - 1q25.3) (29). Later on, Wang et al. (30), using the same strategy with Gao et al. analyzed two Chinese Han families identifying a region on chromosome 19q13 containing about 200 Refseq genes. By Sanger sequencing, Wang et al. found two different one-nucleotide deletions not found in 200 healthy controls in *PSENEN*, encoding for presenilin enhancer (PEN2). As *PSENEN* encodes for one of the four subunits of γ-secretase complex (31), they sequenced all γ-secretase genes in four families and found 1 frameshift mutation in *PSEN1* (14q24.2) and 3 in *NCSTN* (1q23.2). Notably, each family presented a different mutation and all the mutations caused haploinsufficiency of one γ-secretase following the non-sense mediated decay (NMD) of their mRNA. Since γ-secretase catalyzes the intramembrane proteolysis of Notch receptors (30), deficiency of which caused histological features of HS in several mice models (32–34), Wang and collaborators concluded that HS is the results of an attenuated Notch signaling in the skin of patients with *NCSTN, PSENEN*, and *PSEN1* inactivating mutations (30).

A DNA variant affecting splicing was found later by Liu et al. (35) in the family analyzed by Gao and collaborators thus confirming the association of *NCSTN* mutations (and the chromosome region 1q23.2) with HS. *NCSTN* and *PSENEN* novel mutations segregating with the trait were found in families from UK (36), France (37), Japan (38) and one African-American family from the United States (39).

Interestingly, two studies on sequentially recruited patients showed that very few “sporadic” patients, i.e., patients that did not report a family history for HS, presented pathogenic DNA variants in the three morbid genes (40, 41). Deep sequencing of *NCSTN* was performed by Liu et al. (42) on 95 European and African-American HS patients enrolled in the Pioneer I and II clinical trials. The majority (*n* = 57) of patients had a family history of the disease but only one patient with a nonsense mutation (rs387906896; p. R117X) and one sporadic patient with a missense variant (rs147225198; p. A410V) were found, thus reinforcing the idea that mutations in γ-secretase genes are responsible for a small percentage of HS cases and are not sufficient alone to explain all HS phenotypes.

Reduced penetrance of *NCSTN* mutations has been shown once in a Japanese family analyzed by Nomura et al. (43) where the proband's 70-year-old sister carrying the missense variant p.Q568X had never manifested any sign of the disease probably because, unlike to the other affected family members, she claimed to have never smoked.

To date more than 30 mutations have been described in *NCSTN* in HS patients (44, 45), 15 mutations in *PSENEN* (46–48) and only one “likely pathogenic” mutation in *PSEN1* (44).

Interestingly mutations in *PSENEN* results in 3 different phenotypes: (1) HS, (2) Dowling-Degos Disease (DDD), or (3) HS and DDD (47, 49), whilst DDD is not associated with any mutations in *NCSTN*.

Even if the common idea is that HS is the result of a deficient NOTCH signaling in patients with mutations in γ-secretase genes, this claim has been weakened lately by different findings.

For instance, the “likely pathogenic” mutation PSEN1 c.725delC was shown to increase, not to diminish, NOTCH signaling in zebrafish (50). In addition, genomic variations in *TSPEAR* that decrease NOTCH signaling similarly to γ-secretase mutations, have been associated to a novel form of ectodermal dysplasia affecting tooth and hair follicles without any sign of skin inflammation typical of HS (51).

The mechanism by which *NCSTN, PSEN1*, and *PSENEN* mutations lead to HS has yet to be elucidated. This seems a rather complex mechanism as γ-secretase has more than 100 identified substrates (31, 52) and process 21 Receptor Tyrosine Kinases (RTKs) involved in important cellular processes such as cell cycle, survival, differentiation, and migration (53). Gamma-secretase deficiency could also regulate inflammation as it processes important cytokines receptors such as IL-1β R1/R2 and IL-6R (31).

### Genetic of the HS: Other Genes

**Figure 1:**
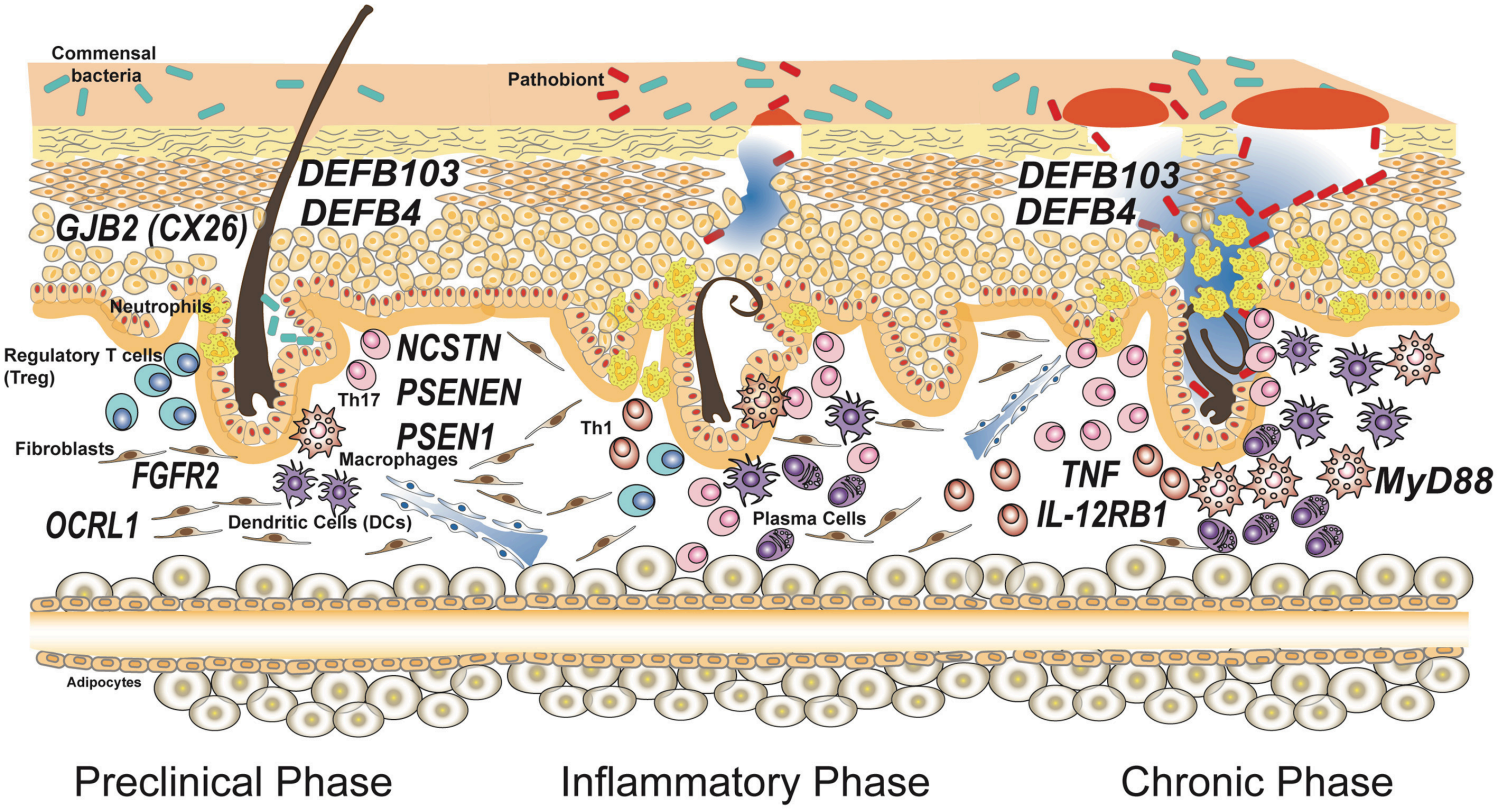
Genes associated with susceptibility and progression of hidradenitis suppurativa. Susceptibility to the disease is caused by mutations in genes involved in keratinocytes homeostasis having a role in maintaining the integrity of the epithelial barrier. Common polymorphisms in genes encoding for proteins involved in the immune response have been associated to severity of the disease and influence the inflammatory and chronic phases. The disease model depicted is based on the most accepted model reported by Berna-Serna and Berna-Mestre (54) for hidradenitis suppurativa.

As shown in Table 1 and depicted in Figure 1, in addition to the 3 genes that encode for the subunits of γ-secretase complex, other 8 genes are involved in HS.

**Table 1:** Summary of the genes involved in HS pathogenesis, including their encoding proteins, functions, and mutation category.

**Gene**	**Encoding protein**	**Function**	**Mutation category**
*PSENEN*	Presenilin enhancer protein 2	Essential subunit of the gamma-secretase complex, an endoprotease complex that catalyzes the intramembrane cleavage of integral membrane proteins such as Notch receptors, and Amyloid-beta Precursor Protein	Frameshift, nonsense, splicing, missense
*PSEN1*	Presenilin 1	Catalytic subunit of the gamma-secretase complex, an endoprotease complex that catalyzes the intramembrane cleavage of integral membrane proteins such as Notch receptors, and Amyloid-beta Precursor Protein	Frameshift
*NCSTN*	Nicastrin	Essential subunit of the gamma-secretase complex, an endoprotease complex that catalyzes the intramembrane cleavage of integral membrane proteins such as Notch receptors, and Amyloid-beta Precursor Protein	Missense, nonsense, frameshift, splice site
*GJB2*	Gap junction protein beta 2, Connexin-26	Member of the gap junction protein family specialized in cell-cell contacts that provide direct intracellular communication.	Missense
*FGFR2*	Fibroblast growth factor receptor	Member of the fibroblast growth factor receptor family that plays an essential role in the regulation of cell proliferation, differentiation, migration, and apoptosis, and in the regulation of embryonic development	Missense
*OCRL1*	Inositol polyphosphate 5-phosphatase	Involved in regulating membrane trafficking and primary cilium formation	Missense
*TNF*	Tumor necrosis factor	Multifunctional proinflammatory cytokine involved in the regulation of a wide spectrum of biological processes including cell proliferation, differentiation, apoptosis, lipid metabolism, and coagulation	Non coding variant that is associated with gene expression
*IL-12Rb1*	Interleukin-12 Receptor Subunit Beta-1	IL-12/IL-23 pathway. IL-12 is implicated in the differentiation of the Th-1 immune response and IL-23 is mediating T17 response, the latter priming chronic neutrophils influx	Missense
*DEFB103*	Defensin beta 3 (hBD3)	Play an important role in innate epithelial defense	Copy number variation
*DEFB4*	Defensin beta 2 (hBD2)	Play an important role in innate epithelial defense	Copy number variation
*MYD88*	Myeloid differentiation primary response protein MyD88	Plays a central role in the innate and adaptive immune response and it is involved in the Toll-like receptor and IL-1 receptor signaling pathways	Nonsense

Mutations in the connexin-26 gene (*GJB2*) on chromosome 13q11-q12 GJB2 gene, that encodes connexin-26 (Cx26), have recently been linked to HS. Mutations in this gene caused Keratitis-ichthyosis-deafness (KID) syndrome, a rare congenital disorder of the ectoderm that gives rise to keratitis, erythrokeratoderma and neurosensory deafness. HS has been reported in association with KID syndrome in a few cases with distinct Cx26 mutations such as D50N, A40V, G12R (55–57).

Cx26 is one of the main connexins in human skin and is normally restricted to hair follicles and eccrine sweat glands (58).

The mutations of Cx26 disturb the gap junctions, specialized channels that connect the cytoplasm of adjacent cells. These cellular structures are important for tissue homeostasis, growth and development and for cellular response to external stimuli (59).

The exact correlation between HS and Cx26 mutations and the interplay of gap junctions and inflammation remain to be elucidated; it is believed that HS might result from the hyperproliferative tendency of KID syndrome patients' epidermis, leading to follicular plugging, cyst formation, and rupture and spillage of keratin and glandular secretions into the subcutaneous tissue, causing an inflammatory response (55).

Recently, Higgins et al. (60) identified a germline missense mutation in fibroblast growth factor-receptor 2 (*FGFR2*) gene in exon 5 (c.G492C, p.K164N) in a patient with HS. *FGFR2* is normally expressed in keratinocytes, hair follicles and sebaceous gland. It is a tyrosine-protein kinase that plays an essential role in cell proliferation, differentiation, migration, and apoptosis, and in the regulation of embryonic development (61). Unfortunately, to date there are no functional and expression studies about this mutation. A predictive analysis with the help of several prediction algorithms has assessed that this mutation may have a pathological consequence on the impaired protein function. Considering that FGFR2 mutations are also associated with acne and that FGFR2 results in the activation of the HS-related PI3K/Akt pathway (caused by mutations in γ-secretase genes), exploration of this aspect could be relevant (62, 63).

Marzuillo et al. (64) identified mutations in inositol polyphosphate-5-phosphatase 1 (*OCRL1*) gene in HS patients. *OCRL1* encodes an inositol polyphosphate 5-phosphatase and is involved in regulating membrane trafficking and primary cilium formation. Mutations in *OCRL1* are associated with Dent disease 2 (DD2), a disorder characterized by proximal tubule dysfunction. In a case report Marzuillo et al. described 5 DD2 patients with *OCRL1* mutations and 4 of these patients were diagnosed as having HS.

Mutations in *OCRL1* drastically reduce the OCRL1 activity, causing an increase of phosphoinositol-4,5-bisphosphate (PI(4,5)P2) levels in the plasma membrane, a substrate of this enzyme. The correlation between HS and DD2 could just be due to an accumulation of PI(4,5)P2, able to increase susceptibility to cutaneous infections.

Considering evidence suggesting the central role of deranged immune response in the pathogenesis of HS, several genetic studies have focused the attention on genes encoding for protein of immune response.

In this context, Savva et al. (65) decided to investigate SNPs in tumor necrosis factor (*TNF*) and Toll-like receptor 4 (*TLR4*) genes, in DNA from 190 patients and 84 healthy controls. They found that only one SNP of the promoter region of the *TNF* gene (-238 TNF gene polymorphism) is related both with susceptibility to HS and with the natural course of the disease; in fact, it is related to more frequent exacerbation and more severe disease. Regarding *TLR4* SNPs, they failed to identify the impact of these SNPs on susceptibility to HS (65).

Indeed, Giatrakos et al. (66) have hypothesized that the dysregulation of antigen-presentation could play a role in the pathogenesis of HS, in particular the IL-12/IL-23 pathway. Considering that both IL-12 and IL-23 receptors have a common subunit encoded by the *IL-12Rb1* gene and that there is an association between this gene and several autoimmune disorders, they decided to investigate the association between the risk for developing HS and SNPs in *IL-12Rb1*. Studying DNA from 139 patients and 113 healthy controls, they observed that SNPs in *IL-12Rb1* did not seem to play a role in the genetic predisposition; however, they found that these SNPs impacted considerably on the clinical phenotype of the disease; in fact, they are associated with more severe disease, extended skin involvement and earlier disease onset (66).

Of note, few times genetic findings contradicted common concepts in HS pathogenesis. This is true, for instance, for the study of copy number variation (CNVs) of β-defensin genes *DEFB103* and *DEFB4* (67). The idea that HS is caused by uncontrolled growth of skin microflora or by a bacterial pathogen colonizing the skin of the patients is testified by the common use of antibiotics as a first line treatment for the disease. Thus, researchers would have expected a deficiency in antimicrobial peptides production, but Giamarellos-Bourboulis and collaborators showed that an increased number of *DEFB103* and *DEFB4* genes, associated with augmented expression of β-defensin 2 and 3 proteins, is an important risk factor for HS susceptibility. However, patients with more copies of these genes were protected against a severe phenotype in terms of both age of initiation and number of affected sites (see Figure 1).

Recently, Agut-Busquet et al. (68) observed an association of Myeloid differentiation primary response gene 88 (*MYD88*) SNPs and susceptibility to severe HS, analyzing the DNA of 101 HS patients. This gene encodes a cytosolic adapter protein that plays a central role in the innate and adaptive immune response. This protein is involved in the Toll-like receptor and IL-1 receptor signaling pathway in the innate immune response (69). Agut-Busquet et al. found a significantly increased risk of developing severe HS (Hurley III) for the GG genotype of rs6853 in *MYD88* gene.

### Genotype-Phenotype Correlation

Different authors have attempted to clinically classify HS in order to stratify patients for clinical trials and identify subpopulations prone to respond to specific therapies. Canoui-Poitrine et al. (70) identified 3 subtypes of disease (“axillary-mammary,” “follicular,” and “gluteal”) by means of a latent class analysis on prospective clinical data of 618 consecutive patients, while 6 different phenotypes (regular type, frictional furuncle type, scarring folliculitis type, conglobata type, syndromic type, ectopic type) were suggested by Van der Zee and Jemec (71). Despite these efforts to distinguish different clinical categories of HS, establishing a clear genotype-phenotype correlation is not possible to date. However, several mutations affecting the components of the inflammasome cascade or the proteins that regulate inflammasome function have been described in syndromic HS patients. The two main syndromes including HS as a part of their cutaneous manifestations are PASH, a disorder presenting with the triad pyoderma, acne and HS (72–76), and PAPASH, a syndrome described by our group and characterized by the same triad of PASH and pyogenic arthritis (77) in whom genetic studies evaluating exons 10 and 11 of the *PSTPIP1* gene revealed a p.E277D previously unreported missense mutation.

PASH patients are generally young adults with a very early onset of the clinical manifestations of the syndrome, especially acne (72–74, 78, 79). For the first two reported PASH cases, it was hypothesized that the presence of alleles with a higher number of CCTG motif repeats close to the *PSTPIP1* promoter deregulated PSTPIP1 expression and predisposed to neutrophilic inflammation (72). This microsatellite may, therefore, be involved as a modifier gene, although it is probably not causal (80). The initial hypothesis was that PASH is a monogenic disorder, but nowadays its polygenic autoinflammatory nature has been confirmed (74, 81). An observational study of five PASH patients (74) showed that their nine gene mutations had already been entered in the database of single nucleotide polymorphisms and that seven were in the registry of hereditary autoinflammatory disorder mutations. Four of these five patients had genetic alterations typical of monogenic autoinflammatory diseases, and the only patient without any genetic changes had Crohn's disease, which is regarded as an autoinflammatory disease. Indeed, mutations of the *MEFV* (Mediterranean fever) gene have previously been associated with the typical clinical picture of recessive familial Mediterranean fever (FMF) and mutations of the *NOD2* (nucleotide-binding oligomerization domain-containing protein 2) gene are associated with an increased risk of developing Crohn's disease (82). A loss-of-function mutation in the *NCSTN* gene has been reported in one PASH patient (79). The nature and location of this mutation do not distinguish it from the reported HS mutations (83), thus supporting a close relationship between isolated HS and PASH.

## Transcriptomics: Differential Gene Expression in HS

The impact of genetics in the susceptibility to hereditary and sporadic HS is not only limited to mutations impairing proteins known to be associated with the disease (i.e., those involved in the γ-secretase pathway); other genetic variations such as epigenetic changes, or variations in regulatory regions could play a role in HS susceptibility or in HS clinical phenotype modulation.

With this purpose, several studies analyzed the gene expression profiles in different anatomical districts (i.e., lesional skin, peripheral blood) of HS patients aimed at discovering novel actors possibly involved in the diseases or in its clinical modulation (see Table 2).

**Table 2:** Overview of gene expression in lesional and non-lesional skin of HS patients, healthy controls, and subjects suffering from other skin diseases, such as psoriasis and atopic dermatitis.

**Gene**	**Expression**	**Tissue**	**Technique**	**Number of subjects**	**References**
Whole genome	50 probes differentially expressed (no validation), 10 putative disease-related pathways	Lesional skin, non-lesional skin whole blood	Affymetrix GeneChip. NO VALIDATION	27 (17 HS patients, 10 healthy donors)	(84)
Drosha, DGRC8, Dicer Exportin-5	Drosha ↓, DGRC8 ↓ in non lesional skin	Skin lesions and non-lesional skin	RT QPCR, IHC	28 (18 HS patients, 10 healthy controls)	(85)
miRNA-155-5p, miRNA-223-5p, miRNA-31-5p, miRNA-21-5p, miRNA-125b-5p, and miRNA-146	miRNA-155-5p ↑, miRNA-223-5p ↑, miRNA-31-5p ↑, miRNA-21-5p ↑, miRNA-146a ↑, miRNA-125b-5p ↓	Lesional and perilesional skin	RT QPCR	25 (15 HS patients, 10 healthy controls)	(86)
TRBP1, TRBP2, PACT, AGO1, AGO2, metadherin, SND1	TRBP1 ↓, PACT ↓, AGO1 ↓, AGO2↓, SND1 ↓	Lesional skin, peri-lesional skin psoriasis, healthy skin	RT QPCR	38 (18 HS patients, 10 psoriasis patients, 10 healthy controls)	(87)
IL-12, IL-23, IL-17	Il 12 ↑, IL17 ↑, IL-23 ↑	Lesional skin, healthy skin	RT QPCR, IHC	18 (10 patients with HS, 8 healthy controls)	(88)
IL-22, IL-20, IL-17A, IL-26, IFN-γ, IL-24, IL-1β, hBD1, hBD2, hBD3, S100A7, S100A8, S100A9	IL-22 ↓, IL-20 ↓, hBD1 ↓, hBD2 ↓, hBD3 ↓, S100A7 ↓, S100A8 ↓, S100A9 ↓	HS lesional skin vs. Psoriatic and atopic dermatitis lesional skin	RT QPCR	37 (8 healthy controls; 14 Psoriasis patients; 7 HS patients; 8 patients with atopic dermatitis)	(89)
IL-1β, IP-10, RANTES, hBD1, hBD2, hBD3, S100A7, S100A8, S100A9, RNAse7	IL-1β↑, IP-10↑, RANTES ↑, hBD1↓, S100A7↑	Keratinocytes isolated from hair follicles	RT QPCR	–	(90)
IL-17, IL-1β, TNF-α, NLRP3, IL1β, IL18	IL-17↑, IL-1β↑, TNF-α↑, NLRP3↑, IL1β↑, IL18 ↑	LESIONAL, non-lesional skin, uninvolved skin from the same patients.	RT QPCR, FC, enzyme-linked immunosorbent assays	54 (44 HS patients, 10 healthy controls)	(20)
IL32	IL32 ↑	Lesional skin and serum	RT QPCR, IHC, ELISA	36 (20 HS patients, 8 psoriasis patients, 8 atopic dermatitis patients)	(91)
IL36	IL36 ↑	Lesional skin and serum	RT QPCR, IHC, ELISA	38 (25 HS patients, 6 psoriasis patients, 7 healthy donors)	(92)
TLR2	TLR2 ↑	Skin lesions, CD68+ macrophages, CD209+ DCs	RT QPCR, IHC, FC	16 (9 HS patients, 7 healthy controls)	(93)
hBD3, RNAase 7, psoriasin (S100A7), dermicin (DCD)	hBD3 ↑	Lesional skin, healthy skin	RT QPCR	93 (36 HS patients 57 healthy controls)	(94)
GSE72702 expression profile of genes encoding sphingolipid-related enzymes from Gene Expression Omnibus database	Perilipin 1 ↑, S1P (sphingosine-1-phosphate) ↑, SMase, (sphingomyelinase) ↑; CerS2 (Ceramide synthase 2) ↓, SK2 (sphingosine kinase) ↓, SPT (serine palmitoyl CoA transferase) ↓	Skin inflammatory lesions, skin biopsies of healthy controls	*In silico* Microarray repository NOT VALIDATED	30 (17 HS patients; 13 healthy skin tissue)	(95)

*↑, up-regulated in HS lesional skin; ↓, down-regulated in HS lesional skin*.

### Whole Genome Expression

To the best of our knowledge, the most complete gene expression profiling in HS patients has been performed by Blok et al. (84), who analyzed lesional skin and whole blood from 17 HS patients comparing their whole gene expression profile with 13 samples of healthy skins (from non lesional areas of HS patients) and whole blood from 10 healthy donors. The authors studied the whole genome expression using the Affymetrix GeneChip HT HG-U133+PM Array (Affymetrix, Santa Clara, CA, US). The first interesting finding is that no differences in NCSTN, PSEN1, and PSENEN gene expression have been found either at skin level or in whole blood from patients and controls. Blok et al. claim that the absence of differences in whole blood between HS patients and controls should be related to a possible post-transcriptional negative control of cytokines production due to augmented serum level of tumor necrosis factor (TNF)-α as reported by Matusiak et al. (96).

When considering HS patients skin, Blok et al. identified 50 probes differentially expressed between lesional and non-lesional skin of HS patients as well as 10 pathways possibly involved in the disease (97); these pathways are (in order of statistical significance based on *p*-values): Granulocyte adhesion and diapedesis, agranulocyte adhesion and diapedesis, atherosclerosis signaling, hepatic fibrosis, primary immunodeficiency signaling, communication between innate, and adaptive immune cells, dendritic cell maturation, complement system, systemic lupus erythematosus signaling and leukocytes extravasation signaling.

The authors, in our opinion, did not exhaustively explain the findings obtained, just justifying the differences in gene expression based on the genetic background of HS patients. However, it should be underlined that Blok et al. acknowledged the limitation of their study related to the relatively small number of samples analyzed and overall to the lack of validation (both immunohistochemistry on *in situ* hybridization as well as RT-QPCR).

### miRNA Regulatory Elements Expression

Another important aspect of gene expression regulation has been widely considered by Hessam et al. (85–87); in three independent studies, the authors analyzed miRNA expression profiles in inflammatory lesions from HS patients.

In the first study, the authors. (85) assessed, using RT QPCR, the expression of Drosha, Drosha co-factor DGRC8, Dicer and Exportin-5 in skin lesions and non-lesional skin from HS patients, skin lesions from patients with psoriasis and skin biopsies from healthy individuals. By finding a down-regulated gene expression of Drosha and DGRC8 just in non-lesional skin from HS patients, the authors hypothesized an early intervention of these miRNA regulators during the first, clinically and histologically not detectable, stages of inflammation, thus suggesting that when inflammation signs become observable only at that moment Dicer and Exportin-5 are involved.

In the second study (86), the expression of inflammation-related miRNA (namely miRNA-155-5p, miRNA-223-5p, miRNA-31-5p, miRNA-21-5p, miRNA-125b-5p, and miRNA-146) was evaluated through RT QPCR in lesional and perilesional skin of 15 HS patients and 10 healthy controls: the above-mentioned miRNA was shown as differentially expressed in HS patients as compared to controls, leading the authors to hypothesize a function in the modulation of the inflammatory response in the lesional skin of HS patients.

In the third study, Hessam et al. (87) enrolled HS and psoriasis patients as well as healthy controls analyzed the expression profile of RNA-induced silencing complex (98) components (specifically, transactivation-responsive RNAbinding protein-1 (TRBP1), TRBP2, protein activator (PACT) of the interferon-induced protein kinase R, Argonaute RISC Catalytic Component-1 (AGO1) and Component- 2 (AGO2), metadherin, and staphylococcal nuclease and Tudor domain-containing-1 (SND1)), also in this case using RT QPCR, in their inflamed tissues (skin biopsies). The authors concluded, after RISC component comparison between skin biopsies of HS and psoriasis patients and healthy controls, that all RISC components were differentially expressed thus highlighting a possible role in the modulation of skin inflammation in HS patients.

Indeed, the three studies of Hessam et al., also in this case with the limitation of the low number of individuals considered and the lack of information about ethnicity of patients and controls enrolled, possibly accounting for genetic differences, evidenced novel possible biomarkers correlating with local skin inflammation to be eventually considered in the follow-up of HS patients (4).

### Cytokine Expression

Due to their widely accepted role in the modulation of inflammatory processes, cytokine-encoding genes have been extensively studied in the context of HS etiopathogenesis.

Schlapbach et al. (88) analyzed, using RT QPCR and validating their findings with immunohistochemistry, lesional skin of HS patients and compared IL-12, IL-23, and IL-17 gene expression with skin biopsies from healthy controls. The authors observed a specific expression of the IL-23/Th17 pathway in lesional skin, thus evidencing, as expected, a connection between the immune system and the inflammatory phenotype in the HS lesions.

Starting from the observation that IL-22 has been reported as correlated with chronic cutaneous diseases such as psoriasis, Wolk et al. (89) evaluated IL-22 encoding gene expression in HS patients. In their work, the authors showed diminished expression of IL-22 and IL-20, but not of IL-17A, IL-26, IFN-γ, IL-24, or IL-1β in HS lesional skin. Furthermore, a correlation between a shortage of IL-22 and IL-20 and reduced expression of antimicrobial peptides (hBD1, hBD2, hBD3, S100A7, S100A8, S100A9) has also been found in HS lesional skin. Wolk et al. concluded that IL-22, same as for other chronic skin diseases, could be another actor potentially involved in HS etiopathogenesis.

Hotz et al. (90) observed a significant increase in IL-1β, IP-10 secretion, and chemokine ligand 5 (CCL5/RANTES), either constitutively or on pattern recognition receptor stimulations, in keratinocytes isolated from hair follicles of patients with HS.

Using a multitasking experimental approach involving RT QPCR, flow cytometry and enzyme-linked immunosorbent assays, Kelly et al. (20), detected an augmented expression of genes encoding IL-17, IL-1β and TNF-α in biopsies of lesional skin from HS patients when compared to biopsies from non-lesional skin and uninvolved skin from the same patients. Moreover, the authors demonstrated an involvement of the inflammasome platform in HS lesions, being increased the expression of NLRP3, IL-1β, and IL-18. Finally, differential cytokine expression was detected in perilesional and non-lesional skin biopsies, leading the authors to hypothesize the presence of inflammation in HS patients present before the development of clinically evident lesions.

Thomi et al. (91) reported an increased expression of IL-36 encoding gene in skin biopsies and serum from HS patients, highlighting a local and systemic involvement of this cytokine, but the exact mechanism of action of IL-36 in HS pathogenesis has not been suggested.

In another independent study, the same authors (92) observed enhanced IL-32 gene expression in both lesional skin and serum from HS patients when compared to healthy controls or patients suffering from psoriasis and atopic dermatitis. Moreover, Thomi et al. identified the cells producing IL-32, namely natural killer cells, T cells, macrophages and dendritic cells localized at dermal level. The authors conclude that IL-32 could be a potential target for novel drug development.

At last, Jenei et al. (99) suggested after performing protein arrays that not only the microbiota and chemical content of human skin show three main topographical areas (dry, moist, oily/sebaceous), but probably in correlation to this, the immune and barrier characteristics of these topographical regions are also distinct, which can make these skin regions become prone to the development of “region-specific” inflammatory skin diseases, like HS on apocrine gland-rich areas and acne or rosacea.

### Other Differentially Expressed Genes

Hunger et al. (93) aimed at exploring the function of TLR2 in the modulation of the clinical phenotype of HS patients, studies TLR2 encoding gene expression in skin lesions of HS patients. Using a multidisciplinary approach consisting in RT QPCR, immunohistochemistry and flow cytometry, the authors demonstrated an up-regulated TLR2 gene expression in HS patients skin lesions, also identifying CD68+ macrophages and CD209+ DCs as the cells expressing TLR2.

Hofmann et al. (94) published a seminal paper on defensins gene expression in the epithelium of HS patients. The authors analyzed through RT QPCR, the expression of HBD3, RNAase 7, psoriasin, and dermicin antimicrobial peptides encoding genes in lesional skin from HS patients (36 individuals) and skin biopsies from healthy controls (57 subjects). It has been observed a defective RNAase 7 expression (both at RNA and protein levels) in HS patients, while HBD3 expression (both RNA and peptide) was increased in HS patients but not in those with a more severe phenotype (Hurley grade III). The authors suggest that lack of antimicrobial peptide expression could predispose to major susceptibility to infections in skin lesions, while reduced HBD3 expression in severe HS cases could be related to a potential anti-inflammatory role.

Dany and Elston (95) using a microarray-based approach analyzed the expression of sphingolipid-related enzymes in skin inflammatory lesions of HS patients and skin biopsies of healthy controls. The authors observed an up-regulation of genes encoding ceramide and sphingomyelin generating enzymes as well as augmented expression of genes encoding enzymes catabolizing ceramide to sphingosine and those converting ceramide to galactosylceramide and gangliosides. Dany and Elston suggested that, based on the findings obtained and acknowledging the limitation due to the lack of evaluation of the sphingolipids generated by the evaluated enzymes, sphingolipid metabolism is modified in HS lesional skin. This study also suffers the absence of RT QPCR validation of the microarray results.

## Proteomics

Two studies on proteins being involved in HS development have been performed by Blok et al. (97) and Zouboulis et al. (100).

The authors analyzed sera from 17 patients with moderate to severe HS (based on Hurley scale), treated with ustekinumab, a monoclonal antibody directed against IL-12 and IL-23 and approved for the treatment of psoriasis. The clinical trial has been designed to understand if any proteomic marker was possibly involved in the successful (or not) treatment with the drug for 40 weeks follow-up. Blok et al. analyzed 1,129 proteins in the sera of HS patients at the beginning and the end of ustekinumab treatment.

Serum proteomic analysis revealed a different expression of 54 proteins in the 17 HS patients when compared to 10 healthy subjects. These 54 differentially expressed proteins, after accurate pathway analysis, resulted involved in inflammatory processes, cellular signaling related to immune processes and tissues architecture modulation. Moreover, among the 4 patients who achieved a good response after drug administration, all were characterized by up-regulated production of Leukotriene A4 Hydrolase (LTA4H), follicle-stimulating hormone (FSH), luteinizing hormone (LH), and human chorionic gonadotropin (HCG), firstly detected with protein array, then validated by ELISA. No effect of ustekinumab treatment has been observed when considering TNF-α, IL-17A, IL-17F.

At the end of their clinical the authors suggest that treatment with ustekinumab, a drug used for psoriasis, was somehow beneficial for HS patients, also proposing the dosage of LTA4H, together with the clinical evaluation using the Hidradenitis Suppurativa Clinical Response (HiSCR) score, for the prediction of the immunosuppressive drug in patients with mild or severe HS.

This work is of some interest in the field of serum markers possibly associated with HS and its treatment. What is strongly needed to unravel the molecular mechanisms at the basis of HS by means of proteome analysis in lesional, pre-lesional, and healthy skin in biopsies from mild to severe HS patients, as studied in the second preliminary study by Zouboulis et al. (100) in 8 HS patients involved and uninvolved skin and 8 gender-, age-, and skin location-matched female patients. The response to pharmacological treatment could be also considered but the main goal should be depicting what is happening at proteomic level in the skin of individuals with HS. Of course, the identification of serological markers related to the clinical conditions and drugs response of patients suffering from HS is also envisaged, since it is easy to be employed in their routine follow-up.

## Data Integration Skin-OMICs

After several studies tackling HS pathogenesis using a single OMICs approach, the one of Hoffmann et al. (101) finally succeeded to integrate skin/serum transcriptomics and proteomics findings obtained in a limited number of HS patients (*n* = 17) with different degree of disease severity and healthy subjects (*n* = 10). The authors made comparisons between transcriptomic and proteomics profiles present in the main repositories or reported in previous articles (see those described above). This integrated approach, the first to our knowledge used until now to disclose the mechanisms at the basis of HS pathogenesis, provided interesting results and opened a new path to approach this complex disease.

Hoffmann et al. propose, based on integrated OMICs findings a novel pathogenic model for HS consisting of two distinct and subsequent stages, initiation with the well-known follicular obstruction and progression of the disease, being the latter characterized by a strong immune response to microbiota, thus adding a novel actor in HS etiopathogenesis.

The authors hypothesized that the differential genes and protein expression (i.e., enhanced expression of innate immune response, immunoglobulins, complements proteins, augmented interferon signature) could be due to the attempts of the immune system, both innate, and adaptive to react to microbiota present in HS patients skin; this is particularly evident if we consider the role of activated complement proteins in HS patients in the fight against commensal skin bacteria, being the main taxa (identified through literature search and metagenomic analysis) *Porphyromonas* and *Prevotella*. Moreover, it is suggested that the strong involvement of the skin-related immune system is a mechanism already observed in other cutaneous diseases that could share with HS the same immunologic mechanisms of response to skin dysbiosis.

Despite the novel approach used, the study of Hoffmann et al. suffers the important bias characterizing all OMICs studies performed to date: few patients analyzed, lack of correlation and integration with GWAS findings. In fact, the authors did not consider in their interesting integrate approach the genetic findings present in the literature, that could have contributed to identifying genetic causative variants in genes encoding the immune system actors involved in the response to dysbiosis, so missing validation of their findings by triple-checking their results with the genetic findings.

## Conclusions

In this review, we collected all the information concerning the OMICs studies performed on HS patients aimed at unraveling the mechanisms at the basis of the disease or associated to clinical severity and/or the successful response to pharmacological treatment (including biological drugs).

The general picture of the OMICs contribution in the context of HS is not so clear and/or rich of clinical useful information, since most of the studies focused only on one aspect (genome, transcriptome, or proteome) of the disease, enrolling small numbers of patients (this is quite limiting for the genetic studies) from different geographical areas, looking just a few aspects of HS pathogenesis without any integration of the findings obtained or a comparison within studies.

In this sense just two articles [(97, 100): described above] constructively compared the transcriptomic and proteomic profiles of skin and serum from HS patients with previous data present in biological repositories. We do think that this is the right path to be followed to disclose the fine mechanisms at the basis of HS and its clinical course.

**Figure 2:**
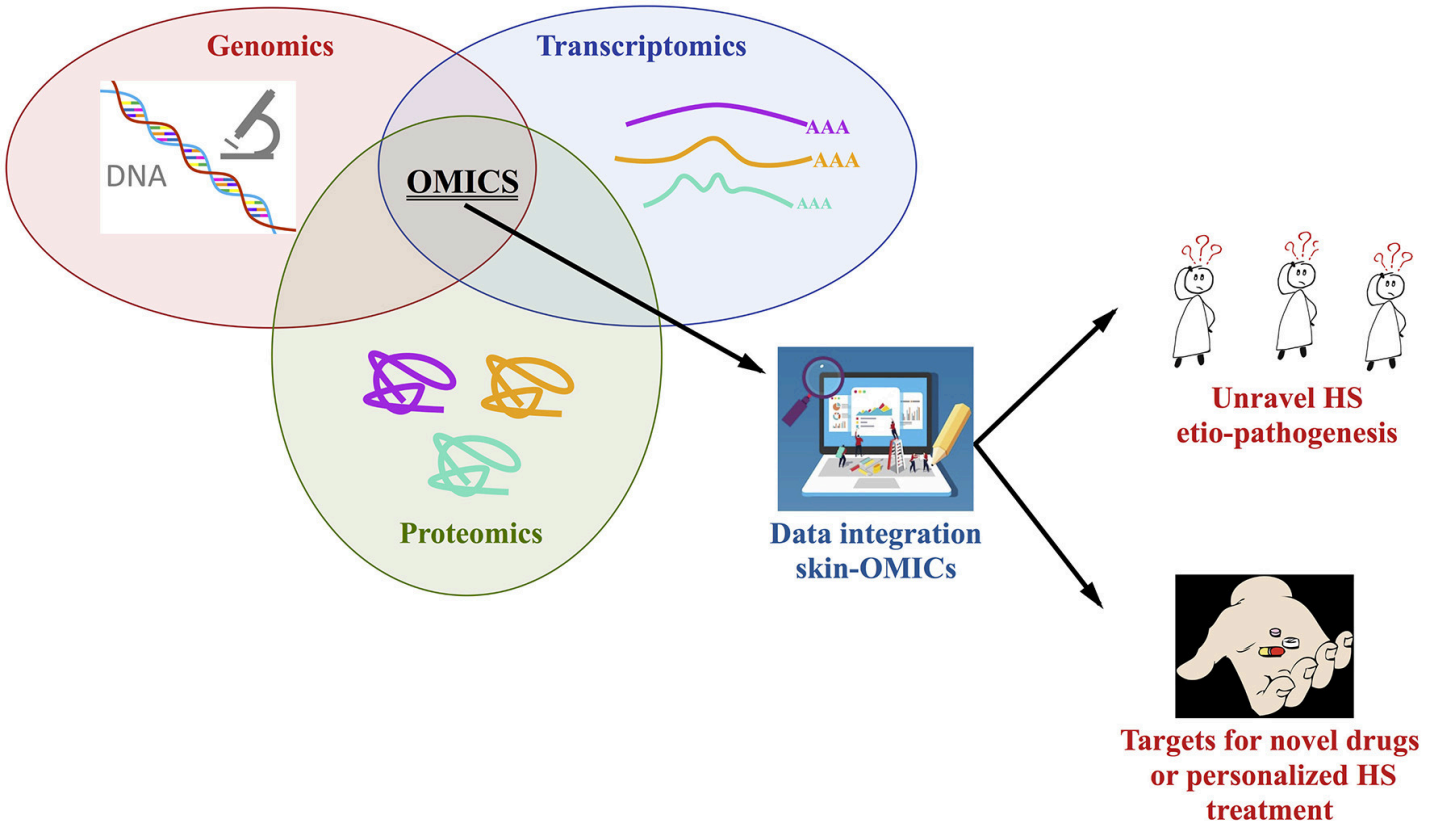
Integrated OMICs pipeline set up for disclosing the actors involved in hidradenitis suppurativa pathogenesis and proposing a personalized treatment for the patients.

An integrated approach using OMICs tools is strongly required to study the full genome, the skin transcriptome and proteome (from lesional, perilesional, and non-lesional biopsies as well as serum) of HS patients stratified based on the severity of the diseases, type of treatment and response to drugs; the number of enrolled patients, with the same ethnic background, is a key issue, especially for the genetic studies, in this sense we do recommend the constitution of consortia to better address this key-point. A comparison and integration with the findings present in the OMICs repositories is mandatory, so in a theoretic pipeline the Skin-OMICs profile obtained from each HS patient should be compared and integrated with repositories and literature data by using appropriate InterOMICs approach (i.e., see the interesting work performed on 16 types of cancer integrating pathways and biological network data by Cava et al. (102). Figure 2 shows the possible integrated strategy to be adopted for tailored diagnosis and treatment of HS patients.

In our opinion, this is the more rapid and robust approach to study the contribution of genome, transcriptome, proteome in the constitution of integrated pathways and networks able to better unravel HS etiopathogenesis, possibly discovering targets for novel drugs design or to personalize HS treatment, in accordance with the new challenges of the precision medicine.

## Author Contributions

PT contributed to the genetics and transcriptomics paragraphs. MB contributed to the genetics of γ secretase paragraph and drew the figures. GG contributed to the genotype-phenotype correlation paragraph and genetic diagnosis. AM and CZ contributed to the design and review of the manuscript and to genotype-phenotype correlation paragraph. SC contributed to the proteome, data integration paragraphs and manuscript design.

### Conflict of Interest Statement

The authors declare that the research was conducted in the absence of any commercial or financial relationships that could be construed as a potential conflict of interest.
